# Quality of Life and Concerns in Parent Caregivers of Adult Children Diagnosed with Intellectual Disability: A Qualitative Study

**DOI:** 10.3390/ijerph17228690

**Published:** 2020-11-23

**Authors:** María Inmaculada Fernández-Ávalos, María Nieves Pérez-Marfil, Rosario Ferrer-Cascales, Francisco Cruz-Quintana, Violeta Clement-Carbonell, Manuel Fernández-Alcántara

**Affiliations:** 1 Department of Health Psychology, University of Alicante, 03690 Alicante, Spain; inmaculada.fernandez@ua.es (M.I.F.-Á.); rosario.ferrer@ua.es (R.F.-C.); violeta.clement@ua.es (V.C.-C.); mfernandeza@ua.es (M.F.-A.); 2 Mind, Brain and Behavior Research Center (CIMCYC), University of Granada, 18071 Granada, Spain; fcruz@ugr.es; 3 End-of-Life Research Network (EOL), 18071 Granada, Spain

**Keywords:** quality life, well-being, welfare, intellectual disability, chronic illness, parents, caregivers

## Abstract

*Background:* Previous studies have confirmed that parenting a child diagnosed with an intellectual disability (ID) can negatively affect the parents’ quality of life in several dimensions. However, fewer have assessed its impact years after the initial diagnosis. The objective of this work was to carry out an in-depth analysis of the current quality of life and concerns of both mothers and fathers of adults diagnosed with ID, having as a reference the moment of the diagnosis. *Methods:* 16 parents of adult children with ID were evaluated using a semi-structured interview format. A thematic qualitative analysis was carried out by employing ATLAS.ti software. *Results:* The results suggested that both the emotional and physical well-being of parents, as well as their interpersonal relationships, had declined. In addition, the multiple life changes that had occurred over the time considered in this study, as well as day-to-day worries, had prevented improvements in their quality of life. *Conclusions:* Several dimensions of the parents’ quality of life were affected years after a child is diagnosed with ID. These included poor physical and psychological health, economic difficulties, lack of social and family support, and lack of time for self-care.

## 1. Introduction

The birth of a child with a developmental disability can have a negative emotional impact and a destabilizing effect on family dynamics [1,2,3,4]. Intellectual disability (ID) is a developmental disorder, which begins before the age of 18 years, and is characterized by cognitive and adaptative behavior deficits, expressed as deficiencies in the conceptual (reasoning and learning), social (social skills, interpersonal communication and emotional intelligence), and practical (daily routines) domains [5]. Some of the risks associated with the diagnosis of ID are related to deficits in verbal memory and in some executive functions such as attention and planning, including the presentation of seizures, vision and hearing disorders, and metabolic diseases [6,7,8].

The diagnosis of ID and its associated physical and emotional symptoms, as well as the changing needs of children with ID, can pose additional difficulties during the parenting process. Previous studies seem to indicate that raising a child with ID can affect the parents in various aspects of their lives such as: (a) Leisure time (because they neglect their own needs and self-care to become constant caregivers) [9,10,11]; (b) employment, (because often one of the parents quit their job to devote more time to the needs of their child) [9,11]; (c) social relationships, (resulting from a perceived lack of support and rejection by their social environments) [9,12]; (d) family dynamics, (because they prioritize caring for their child with ID over other obligations, even diminishing the time they devote to their other children) [11,13]; (e) physical and psychological health, (because most parents experience a high degree of stress and anxiety as well as multiple physical ailments resulting from care provision) [11,14,15]; and (f) relationships with their partners, (which often become neglected due to the high level of attention that their child requires) [9,11,16,17].

Furthermore, various groups have shown the appearance of complex emotions in parents such as sadness, anguish, helplessness, and guilt [17,18,19,20], as well as the feeling that each day is a constant struggle in which they act as supervisors that care for and protect their children [21,22]. In addition, most parents also report experiencing difficulties in their relationships with health care professionals and educators alike who, parents say, often show a lack of empathy and involvement [17,23,24]. In terms of social empathy, some parents say that children with ID are continuously rejected and discriminated against, which in turn, causes them to feel great suffering [9,11,21,25]. They constantly are worried about the uncertainty of the evolution of their child’s disability at a physical level and some other levels like academic performance, or how isolation and social rejection could affect them, [2,23].

Correspondingly, research shows that the physical and emotional well-being along with the quality of life of parents, who care for people diagnosed with ID, tends to deteriorate, especially, in cases of severe ID [26,27,28,29]. According to the World Health Organization (WHO) [30], quality of life is a broad concept that includes factors such as the person’s physical and psychological health, level of independence, social relationships, personal beliefs, values, expectations, and concerns. Moreover, according to Hoffman et al. [31], the quality of life of families with members diagnosed with ID includes dimensions such as family interaction, parenting, emotional well-being, physical/material well-being and disability-related support that must be evaluated. The academic literature indicates that the quality of life of these parents may be affected from the moment of diagnosis of their child with ID, but especially during childhood [32,33]. Besides, some studies have shown that the well-being and quality of life of the parents of adolescents or young adults with ID did not improve over time [2,17,32,33,34,35]. This is likely because of continued fear and concern about the uncertain future of their children. As these parents age both their economic and personal relationship problems keep growing, together with the fact that they spend little time engaged in leisure activities or establishing social relationships [17,34,35]. Likewise, the increased life expectancy of people with ID, means that these parents spend longer time caring for their children, which could negatively affect their physical and psychological health [21,22,36].

Although previous work has indicated a decrease in the quality of life of these parents during the early years after the diagnosis of ID, the quality of life of these parents when their children reach adulthood remains poorly explored. Therefore, the main objective of this work was to analyze the current quality of life and concerns of parents of adult children with ID in the present moment, having as a reference point the time of diagnosis, by collecting information from first-hand accounts from these parents.

## 2. Materials and Methods

### 2.1. Design

This study had a descriptive qualitative design based on a thematic analysis. We followed the proposal by Braun and Clarke [37], to obtain results that could be used to support the appropriate interpretation of these subjects of study. The main research question of the present study was: How does parenting a child with a diagnosis of ID impact the quality of life and concerns of parents?

### 2.2. Participants

Purposive sampling [38] was used by selecting parents whose children were diagnosed with ID, and who received care at a non-profit association located in the province of Granada (Spain). The association offers day-care services for people with ID and families who need specialized support and residence care for adults with difficult sociological situations. The study inclusion criteria were parents who were the main caregivers of a son/daughter aged over 18 years diagnosed with ID according to DSM-5 criteria [39]; and who spent at least 3 days a week with their child.

The final sample comprised 16 participants, 5 fathers (31.25%) and 11 mothers (68.75%), whose age ranged from 53 to 83 years. These parents had been the main caregivers of their children from birth to the time of the interview. The children with ID spent 8 h a day, 5 days a week, in the day-care center, and the rest of the time with their parents. The children who were residents there spent 4-5 full days a week in the center, and the rest of the week with their parents. All the participants in this study had only one child diagnosed with ID. The number of additional children for each parent appears in Table 1.

The age of the children ranged from 18 to 40 years of which 5 had been diagnosed with ID from birth (up until 1 year) and 11 were diagnosed afterwards (from age 1 to 13 years). Four of these children had been residents in the center for up 6 to 8 years and all the children considered in this study were unemployed.

The WHO [40], established four degrees of severity of disability: Mild difficulty (between 5% and 24%), moderate difficulty (between 25% and 49%), severe difficulty (between 50% and 95%), and complete difficulty (between 96% and 100%). To establish the degree of severity, the individual’s ability performance (capacity to perform tasks) was compared and evaluated to the normal expected level by physicians [40]. In this current work, the disability of the children considered ranged between 33% and 99%.

The participants were recruited cyclically and progressively, and so the interviews were conducted at the same time as the data were being analyzed (constant comparison). The sample recruitment ended when theoretical saturation was reached for the main codes and themes described below (i.e., when new interviews no longer added additional codes or relevant information) [38].

### 2.3. Procedure

The care association was contacted, and a meeting was held with its professionals to explain the objective of this work. Next, we scheduled meetings with family members who met the inclusion criteria to inform them about the characteristics of the work, objectives of the study, and to request their collaboration. Family members who voluntarily agreed to participate received a document with information about the study and signed their informed consent to participation. None of the parents approached declined to participate in the study, and none of them were remunerated for their participation. We ensured the confidentiality of the data collected by omitting all names and identifying data by assigning each participant an alphanumeric code. The interviews were conducted from July 2017 to January 2018 at the care association facilities in a medium-sized room with adequate ambient conditions. Only the participants and the interviewer were present in this room. This study was approved by the Human Research Ethics Committee at the University of Granada (Ref: 445/CEIH/2017).

The data were collected through a semi-structured interview (see Table 2) that assessed the experiences of parents in relation to the diagnosis of ID in their children. The questions to evaluate the past and present experiences were open-ended and collected information on the changes that had occurred as a result of the diagnosis, at the family, social, and partner levels. We also examined their experiences as caregivers on a physical and emotional level, as well as their current and future concerns. We wanted to explore their memories of these moments and how these continued to impact their present lives.

All the researchers involved in the study were health psychologists and had previous experience in attending vulnerable populations. One interview was conducted with each participant. These interviews took place in individual sessions and were conducted by one researcher (M.I.F.A.), who had previous training in qualitative analysis and psychological treatment, meaning that the researcher could offer psychological support to participants who needed it, although this was not required in any of the cases reported here. The researcher who conducted the interviews did not belong to the association and had not had previous contact with the participants. The interviews were digitally recorded and later transcribed verbatim using f4 software [41]; its duration ranging from 45 to 90 min.

### 2.4. Data Analysis

Following the proposal by Braun and Clarke [37], we carried out a structured thematic analysis in six main phases. First, the researchers familiarized themselves with the data by transcribing the interviews they had conducted and by repeatedly reading the material. In the second phase of the analysis, we started an inductive coding process (line-by-line) in which the most relevant information was organized into different codes relevant to the subject of this work [42]. The name of these initial codes were created, based on participant’s discourse and previous literature review, by the first author (M.I.F.A.) and was reviewed by two of the researchers (M.N.P.M. and M.F.A.) In the third phase of the study codes that presented similar patterns were integrated into the initial themes. In the fourth phase, a continuous review of the different codes and themes proposed in the preliminary analysis process was carried out, and on several occasions, we went on to recode, propose new codes, generate new definitions, and refine the themes.

During this phase, and to guarantee the objectivity of our analysis, the different codes and themes identified were discussed, analyzed, and approved by consensus among the three researchers involved in the analysis. The analysis process ended in the fifth phase, when theoretical saturation was reached, that is, when the researchers had definitively identified, by consensus, four main themes made up of various codes (see Table 3).

Different strategies were employed to guarantee the rigor and the trustworthiness of the results [43]. The codes and themes were generated through triangulation among three of the researchers (M.I.F.A., M.F.A, M.N.P.M.) to ensure the confirmability [44]. In the results section, a clear distinction was made between verbatim quotations of the participants and interpretations made by the researchers. Regarding reflexivity, none of the researchers had any previous relationship with the association that facilitated the data collection or with any of the participants. In addition, we included several quotations to help readers assess the inferences and interpretations made by researchers [45]. A bracketing technique [46] was used to highlight possible researcher bias introduced during the interviews and data analysis. Finally, the quotations selected for the results section were initially translated into English and then back translated into Spanish to ensure accuracy.

The research team that collected and analyzed the data had previous experience in qualitative research and qualitative analysis. Finally, we produced the report to present our data analysis in relation to the phenomenon under study by using. ATLAS.ti software, version 7.5.4 (Scientific Software Development, Berlin, Germany) [47], was used for the data analysis. Table 3 shows the four main themes that emerged from the analysis along with the main codes used.

## 3. Results

### 3.1. Theme 1: Changes Produced After the Diagnosis

Most families noted that their child’s diagnosis of ID triggered several changes in their family dynamics. Some of them had had to move home, or to a city, to try to find a residence center that would be more comfortable and that would adapt to their child’s disability both now and in the future. Other families had moved to be closer to centers where their child could receive adequate treatment to survive, or to be closer to their immediate family who could help with care provision.

“*I was with my mother at her house for a while with my daughter, until we went to live in an apartment, but my mother said that my daughter couldn’t live there. So, we stayed in the village until we could sell the apartment and buy a house in the village so that my daughter would inherit this place in the future, which would be better*…”.

(I01 Mother)

“*We’ve always wanted to look for solutions, in fact, we moved to a village, because we saw that she was not comfortable with the previous house. [I’m] always thinking about her well-being. We also moved to a new house because we found this center nearby, and when you see that she’s well taken care of, then you relax, of course […]. Also, my mother was a very big pillar [of support], because I was working at that time and my mother was the one who helped me with her*...”.

(I02 Mother)

Although other parents continued to live in the same home, they had also had to modify their home so that their child could live as comfortably as possible, including changing the width of the doors, installing handrails in the hallways, building a room on the ground floor if the home had stairs, or buying an adapted vehicle to transport their child.

“*Everything we have done has been for the good of the child. We have had to buy and change vehicles for the child, for his comfort. He did not want to be in the old apartment, he had many sudden crises, that’s why we bought the land, we did the construction, we changed the house, and we built a room on the ground floor, even a swimming pool, because, as he really likes the water, that was good for him*...”.

(I03 Father)

In addition, all the parents said that one of the members of the couple had had to quit their job in order to dedicate themselves exclusively to caring for their child with ID. In this sense, the parents highlighted that, they had had to request many periods of leave or days off from work in order to attend to complications or the multiple medical interventions their child had had or were receiving at the time.

“*My wife did have to quit her job, and she had a fairly good job… We had to weigh up between taking care of our son or earning money. And she made even more money than me, but we had to decide, so she had to leave her job and take care of our son*”.

(I11 Father)

“*When I was working, the teachers weren’t able to control my daughter, so I had to come out of work [to pick her up]. Walking, it took me more than half an hour, and then I had to make up the lost hours at work afterwards. Although at work they did give me permission, and everything was fine*…”.

(I05 Mother)

Furthermore, all the parents said that their family’s income had been seriously affected. From the time their child had been diagnosed, not only had they lost or abandoned their job to take care of the child with ID, but also they had had to carry out many medical and psychological interventions to improve their child’s quality of life. These interventions had been quite costly, and some parents had even had to ask for financial aid from their closest family members because they had found it difficult to obtain disability benefits when needed aid. They still had concerns about their finances at the time of the interview because these parents feared leaving their children without enough money to afford a residence where they would be well cared for in the future.

“*When she was discharged, the director told us: Get it into your head your daughter will have to be in a wheelchair [for the rest of her life]. We said that we would do everything possible to prevent this from happening, and so, her mother took her to rehabilitation for 7 years, and during those years, we paid 45,000 pesetas (€270.45) every month. Of course, on some occasions we had help from the grandparents*…”.

(I13 Father)

“*I have the future sorted out with the residence. Yes, I am concerned about money, so what I do is [put some money aside] for him, so that he has money in the future when we are not there and he can pay them, but of course, it is complicated because we don’t earn that much, and so that worry is there*...”.

(I04 Mother)

Half of the parents had observed a change in their family relationships with their other children (with the siblings of the child with ID). This was because they had had to stop being children or siblings from the time of the diagnosis so that they could become another caregiver, and this had caused them sorrow or sadness. However, the other half of the parents said that the siblings of the child with ID had helped them from the beginning without any problems, and had even gone as far as to give up certain trips with their friends in order to spend time with their sibling with ID. They said that their other children assumed that, in the future when their parents are gone, they would take over this caregiving role.

“*Her brother had to grow up, and he was only 4 years old. He has always had to live with that nightmare, and that my 24 h of attention were directed to my girl. They have a good relationship as siblings, but I think he did feel jealous, and still does nowadays. […] He lived through it in sorrow*...”.

(I14 Mother)

“*Her brother, delighted as always, sits down and listens to her. When he has a moment, he comes and sits with her and he loves it, and he laughs with her. He likes it when she tells him what she is and isn’t doing. He spends his time with her, and he loves it, and she already feels so integrated and so good too. Since he was little, he’s spent a lot of time with her, he even preferred doing that to going out with his friends. Today I can rest and assure that if something happens to me, I know her brother will be there with her*”.

(I06 Mother)

### 3.2. Theme 2: Interpersonal Relationships

Most parents said that they had not received enough family support since the diagnosis of their child, and that they perceived a lack of understanding, empathy, and even a certain rejection of their child with ID. Parents felt a lot of sorrow because they had been distanced from their closest loved ones. However, that sorrow had intensified when they thought about the future of their children, because they worried that their child would be alone when they were no longer alive.

“*It’s that, they all say they love him a lot, but I feel that they’re not being honest. And I have my daughter, and she says that we should solve our own problems, but I already have enough with mine […]. In the village, I used to say, let his cousins come over and play with him and he can learn things, but his cousins have their own lives and they don’t think about coming to see their cousin or to help their aunt*…”.

(I10 Mother)

“*Her brother has lived his life, but he has not been caring. That still bugs me, because he says he’s going to take care of her, but I won’t be here to see it, and that scares me. I’ll believe it when I see it, right? That’s my way of thinking*...”.

(I07 Mother)

Moreover, all the parents stated that their social relationships had suffered both at the time of diagnosis and at the present time. They had sometimes been involved in confrontations as a result of the anger they had felt when they saw their social circle had moved away from them because they did not have the same family dynamics as other families without children with ID.

“*We don’t have anyone; I don’t know if this happens a lot in these situations. With any help or simply being able to speak to someone we would be very grateful, but when people see our situation, they move away, they continue their lives, they don’t have the same life as you and they don’t empathize with you, it is easier to run away*…”.

(I15 Father)

“*What friends? You have no friends; nobody wants to listen... If you aren’t well, nobody wants to be by your side... Before, when I had to ask for help, it was when the children were boys and I was alone because my husband was working, and I had a friend who, from that moment, became very distant, we barely speak to each other now*...”.

(I16 Mother)

Also, approximately half of the parents said that their relationship with their partner had deteriorated from the time of the diagnosis of their child until the present day. They said that since then, they had dedicated their time exclusively to being caregivers, to improving the life of their child, and had completely abandoned their relationship. They also said that they argued more because of a lack of communication about educating their child. However, the other half of the parents said that that the diagnosis of their child had not negatively affected their relationship with their partner, but on the contrary, it had made the bond between them stronger. Nonetheless, all the parents said that, despite all the difficulties and obstacles they had experienced, they had continued forward in their marriage.

“*We don’t go out, we don’t have a life, because we have to be with him, and we are locked up 24 h a day, the time he’s here in the center, is the only free time we have. Of course, we can’t have a married life, we’ve been like this for 37 years. And we have more and more arguments*…”.

(I03 Father)

“*The usual thing when there is a problem of this type is separation, but it united us more, it united us a lot, because we believed that the person who was really in need was him, not us, and so we dedicated ourselves to him, and that dedication made us unite more*…”.

(I11-Father)

### 3.3. Theme 3: Physical and Emotional Well-Being as a Caregiver

Most of the parents confirmed that their well-being in terms of health had not improved in the adult stage of their children. In fact, they had perceived a great deterioration over the years from the moment of diagnosis. Regarding physical health, parents had noticed being very worn down physically: This was characterized by excessive tiredness, which made it difficult for them to carry out their daily tasks. These parents also said that their sleep schedule had been affected, that they had problems falling asleep because of their day-to-day concerns about their children, or they felt sleep-deprived because their child with ID required such continuous care that they had to wake up several times throughout the night (see Table 4).

In relation to emotional well-being, all the parents reported the constant appearance of the symptoms of anxiety, mainly associated with the health, social, and/or personal complications that their child had at any given moment.

In terms of leisure time, most of the parents said they felt distressed about not having any time for self-care or personal enjoyment because they did not have enough social or family support for the care of their child. Consequently, they played continuous roles as caregivers and had forgotten about leisure time as individuals or a couple.

These parents also claimed to have learned to live with their child’s diagnosis. However, they pointed out the difficulty of fully accepting the diagnosis of their child, as well as its implications, and reported having had recurring feelings of sorrow, grief, or sadness.

Most of the participants said that their child with ID had also positive effects on their lives, especially, by helping them to live more intensely day by day, They said that their child had brought them an incomparable special affection, which compared to their thoughts at time of the initial diagnosis, they now thought that this was the best thing that could have happened to them.

### 3.4. Theme 4: Concerns about the Future

**Figure 1:**
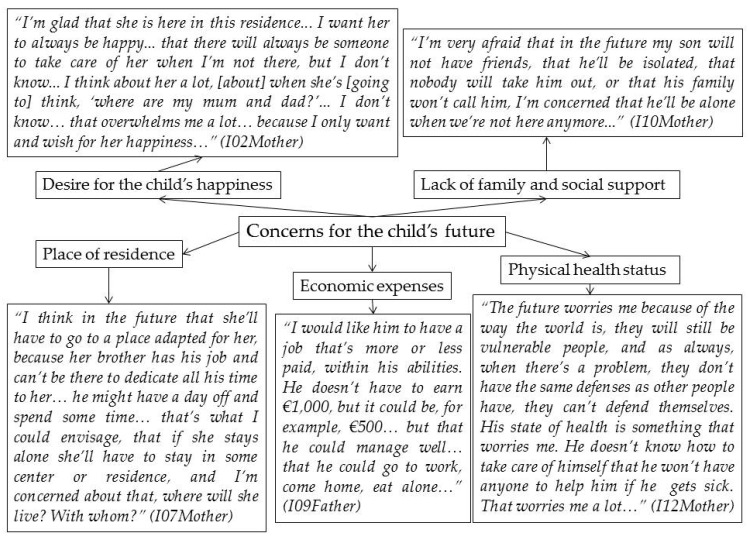
Quotations in Theme 4: Concerns about the future.

All the parents constantly thought about the future of their children. In particular they thought of the time when they being their main caregivers eventually pass away. One of their concerns was related to their child’s future residence. The parents thought that living in a residence or having a family member who would adopt the role of caregiver would be the best options. However, they had their doubts and uncertainties that any of these options would be fulfilled, which led them to feel fearful (see Figure 1).

Secondly, these parents were also concerned that their child would have financial expenses. Parents worried their children would not be able to get by because they would not have a job, a caregiver to help, or because they did not understand how to manage money.

The physical health status of their children had also been a priority for these parents from the time their child with ID had been born. Parents said that, from the time of the diagnosis of ID, their children had had several kinds of treatment to help maintain a good physical health and even to improve it as far as possible, but that they were concerned that in the future when their child would be alone, that they would not know how to take care of themselves, their quality of life would worsen, or they would get sick.

Too, all the parents said that the thought of their child lacking family support or becoming socially isolated when they are were gone worried them and it caused them to feel intense fear. They strongly remembered the feelings of loneliness and rejection that they had felt from their immediate closest friends and family and did not want their children to experience that feeling.

Their desire for the happiness of the child with ID was the main and constant goal of all these parents, both for their present and their future. Thus, on many occasions they had thought that their child would not be happy when they were no longer around, and this had made these parents worry and feel sorrow, because they believed that no one would be able to offer their child the same affection that they had given them.

## 4. Discussion

The objective of this work was to analyze the current quality of life and concerns of the parents of adults diagnosed with ID, having as a reference point the moment when they receive the diagnosis of their child. Our findings show that the changes and the physical and psychological impacts produced at the time of the diagnosis of their child with ID were maintained and in some cases they even intensified. Additionally, we identified important difficulties that these parents had experienced in many dimensions of their quality of life, as well as several concerns they had about their child.

Previous research has shown that the lives of parents undergo important changes after the diagnosis of ID in their child and that not only these changes affect their social, family, work, and personal lives, they also negatively impact their physical and mental health [9,10,11,12,13,14,15,16,17]. The results of this current work confirm these changes, and furthermore, show that from the time of the diagnosis, the main objective of these parents bad been to implement measures designed to improve the well-being of their child.

Based on the proposal of Park et al. [48], we can speak of both individual and family quality of life. The former includes feelings of well-being, positive social participation, and opportunities to reach personal potential. Similarly, another definition proposed by the WHO [30] includes the mental and physical health of the person and considers, factors such as autonomy, social relationships, beliefs, thoughts, values, and personal concerns. The dimensions of quality of family life include satisfaction of family needs and the possibility of family members enjoying being together, and being able to participate in activities that are important to them [48]. Family quality of life has been studied in depth in families comprising at least one member diagnosed with a disability [31,49] and five key dimensions, which we will use to discuss the results of this present research, have been identified [31]; (a) family interaction (relationships between family members); (b) parenting (activities carried out by relatives to help children grow and develop); (c) emotional well-being (aspects that address the emotional needs of the family); (d) physical/material well-being (aspects that address the physical/material needs of the family); and (e) disability-related support (the informal and formal support received that benefits the family and family members with a disability).

First, regarding family interactions, in agreement with findings by previous researchers [50,51,52,53,54], our results indicate that, the role of the siblings of the person with ID had usually changed. Siblings also tended to have become caregivers, which had a positive effect on their parents because it had reduced the individual burden of care that they had had to carry. However, this also meant that these siblings had received less attention from their parents because of the more demanding needs of the child with ID. This can generate emotional problems in family dynamics, communication, and functioning, as indicated in previous research [50,55,56]. In this sense, interventions such as “Triple P Positive Parenting” programs, which have been shown to improve parental skills, could help improve the adjustment and interactions in these families [57].

Second, we found that raising a child with ID was accompanied by high levels of psychological distress, related to concerns about the course of the child’s disease and the multiple changes that can arise from the diagnosis of the ID [58]. The search for solutions and professional support were some of the strategies used by parents to adapt to the new situation [3,59,60] and the use of such strategies has been shown to have a buffering effect on the caregiver’s burden [61,62]. Moreover, participating in work activities is another strategy that can help these parents. For example, research conducted by Ha et al. [32] concluded that employed parents who had a child diagnosed with ID, showed lower levels of negative affect and their psychological well-being was higher. However, the results both from this study and from previous research indicate that an overprotective relationship with the child with ID, combined with the large amount of time parents dedicated to the care and supervision of these children, sometimes led them to experience difficulties and prevented them from combining their work life with the care of their child [9,11,21,22].

Third, in terms of emotional well-being, also in agreement with previous research [63,64,65], the parents included in this present study showed signs compatible with a deterioration in health as the result of their continuous efforts to raise their child with ID. The shortage of time available for self-care and leisure, as well as the scarce external help received [56,66], may have been related to the low quality of life of these parents. Nonetheless, spare time is key to maintaining physical and mental health, and to be able to dedicate time for personal enjoyment [59,67,68]. These parents also often experienced emotions related to mourning, such as intense grief, sadness, and sorrow, both at the time of diagnosis and later [69,70]. Sorrow related to their child’s ID also produced an overload process, and negatively influenced the physical and emotional well-being of these patents [71,72]. Also similar to previous research [2,17,23,35,73,74,75,76], another aspect that negatively impacted these parents was their great concern for the emotional, social, and functional development of their children, from the time of diagnosis onwards.

Although the interview questions fundamentally analyzed the most complicated aspects related to the experiences of having a child with ID, the discourse of parents simultaneously expressed some positive aspects related to the care and upbringing of their child. Despite all the difficulties and obstacles they had had to face, they still felt that the birth of their child with ID was the best thing that could have happened to them [11,23,77,78]—a finding which can act as a protective factor [59].

On another note, mindfulness-based interventions and work with acceptance could effectively reduce the stress and depression levels of these parents and so, it can help improve their emotional well-being [79,80]. Likewise, self-care, kindness to oneself, and time spent on leisure and sports are strategies that can help promote the physical and mental well-being of caregivers [59]. Finally, the information provided by health professionals regarding the diagnosis and prognosis of the child with ID, can play an important role in the process of parental empowerment [81].

Fourth, with regard to material resources, we found that at least half of families with children with disability suffered economic difficulties, both because of the decreased income resulting from some parents having to abandon their employment, and the expenses that arose when trying to satisfy the multiple needs of their child [11,27,82,83]. The economic status of these families sometimes forced them to ask for financial support from close friends and family members [84,85]. Furthermore, it has been observed that the greater the financial instability, the greater the detrimental effects on the quality of life of these parents [86]. According to Park, Turnbull, and Turnbull [87], financial problems are related to other consequences such as the family having limited access to medical care, their reduced opportunities for free time, living in insecure neighborhoods, or homes that are unhealthy and not adapted for people diagnosed with ID—all leading to increased stress levels, decreased self-esteem, and problems in family interpersonal relationships, such as conflicts between the parenting couple or related to the upbringing of their children.

Finally, both this research and other studies coincide that parents report having received very little family or social support; they had usually felt abandoned, rejected, and misunderstood by their closest family and/or social circle, which sometimes caused problems between the couple [11,21,25,56,88]. Social support has been described as one of the most important protective factors for maintaining the health and well-being of parents of children with ID: It has been found that those who received formal or informal support were less stressed, more optimistic, and manifested increased levels of well-being and satisfaction with life [56,77,89,90,91,92]. Thus, support programs or social networks for parents and their child with ID can improve family quality of life and help achieve multiple benefits both at the interpersonal and intraindividual levels [93,94]. In this sense, Bray, Carter, Sanders, Blake, and Keegan [95], found that the use of peer support programs by parents with children with ID, reduced the levels of psychological distress these parents suffered and strengthened their ability to cope with bringing up the child.

Our results indicate that the main needs of these parents are related to: (a) Improving the relationship, communication, and functioning between family members to reduce the amount of time they spend delivering the constant supervision required by their child with ID; (b) decreasing the intensity and frequency of their feelings of grief, sadness, and sorrow, their constant worries, and fostering acceptance; (c) making the care of their child with ID compatible with the parents’ job(s) by using formal and/or informal support; (d) helping parents feel more understood and supported by their social and family environment; (e) being more aware of the positive aspects of raising their child.

Thus, future research must be designed to study the effectiveness of counselling and socio-emotional interventions to reduce the psychological distress of these parents and reinforce their ways of coping [80,95,96]. Likewise, it is important to develop diversity and inclusion policies. Therefore, service organizations that serve people with IDs should be actively involved in providing housing or residential support for this population to help alleviate the concern and anxiety parents feel when thinking of the time they can no longer provide the adequate care for their child and to promote the autonomy of the person with ID [96].

Lastly, this study had both strengths and limitations. Regarding its strengths, we carried out an in-depth thematic analysis of the quality of life and concerns of parents of children diagnosed with ID who have now reached adulthood. This helped us to understand their physical and emotional well-being and to identify factors and/or concerns that had disrupted their quality of life. This data was collected through semi-structured interviews using triangulation between researchers to establish the main themes and codes as well as quotations, which increased the trustworthiness of this study. In terms of its limitations, although our sample was representative, providing enough information to study the research question in depth, it was not a large sample. Since the participants came from a single center, the generalization of this data to other populations requires caution. Another limitation was that, so far, no triangulation has been done with the study participants. That is to say, the results have not been shared with them. In addition, this present research analyzed the experiences of parents when their child with ID had already reached adulthood, but did not consider how the quality of life of these parents might have evolved during the different stages of their child’s development. This data would allow us to make comparisons and extract more conclusive results. Besides, the codes and themes were considered statically based on the discourse of the parents. They indicated how the experience of caring for a child diagnosed with ID had changed their lives and recalled the most important events from their personal biographies. However, given the time elapsed from the time of the diagnosis to the interview, it is possible that they had not fully remembered all their feelings or experiences from that time. Also, we did not collect any information in relation to how the child diagnosed with ID moving house affected the parents. Future multidisciplinary studies should also consider other dimensions of quality of life, focusing on physiological or biological biomarkers in this population. Finally, it would also be interesting to carry out studies that explore the differences in the quality of life of parents of children with different levels of ID. Finally, future studies should include the perspective of professionals from diverse disciplines.

## 5. Conclusions

In conclusion, parents of children with ID experienced problems in several dimensions of their quality of life. The most noticeable were changes in their family dynamics and place of residence, effects on their physical and psychological health, loss and/or abandonment of employment, economic difficulties, and a lack of social and family support and time for self-care. Despite originating after the diagnosis of ID in their child, these changes are still present nowadays because these children continued to require care and their parents strived to maintain their well-being by providing them with constant dedicated care, all of which had personal, economic, and social repercussions [74,75]. Notwithstanding, in this study we also found that feeling the affection of their child with ID and having support from their other children were protective factors that helped these parents persevere in their situation.

## Figures and Tables

**Table 1:** Sociodemographic characteristics of the sample.

Participant	Sex	Age	Kinship	Educational Level	Occupation	Marital Status	Offspring Sex-Age	Timing of Diagnosis	Degree of ID	Number of Additional Children	Residence
I 01	Female	71	Mother	Primary	Homemaker	Married	F-39	Post-Birth (2 years)	86%	1	Family
I 02	Female	59	Mother	Primary	Homemaker	Married	F-27	Post-Birth (2 years)	84%	0	Residence
I 03	Male	69	Father	Primary	Retired	Married	M-37	Birth	99%	0	Family
I 04	Female	83	Mother	Primary	Homemaker	Married	M-40	Birth	79%	0	Residence
I 05	Female	71	Mother	Primary	Homemaker	Widow	F-31	Post-Birth (3 years)	65%	0	Family
I 06	Female	63	Mother	Primary	Homemaker	Married	F-36	Post-Birth (10 years)	70%	1	Family
I 07	Female	66	Mother	Primary	Retired	Married	F-32	Post-Birth (10 years)	75%	0	Family
I 08	Female	54	Mother	University	Homemaker	Married	M-18	Post-Birth (4 years)	48%	0	Family
I 09	Male	53	Father	Secondary	Unemployed	Married	M-18	Post-Birth (5 years)	48%	0	Family
I 10	Female	56	Mother	Primary	Employed	Married	M-31	Birth	75%	1	Family
I 11	Male	67	Father	Secondary	Retired	Married	M-39	Birth	98%	0	Residence
I 12	Female	62	Mother	University	Homemaker	Married	M-39	Birth	98%	0	Residence
I 13	Male	72	Father	Secondary	Retired	Married	F-39	Post-Birth (3 years)	86%	1	Family
I 14	Female	65	Mother	Secondary	Freelance	Married	F-36	Post-Birth (2 years)	33%	0	Family
I 15	Male	64	Father	University	Retired	Married	M-27	Post-Birth (13 years)	75%	1	Family
I 16	Female	56	Mother	University	Unemployed	Married	M-28	Post-Birth (11 years)	36%	1	Family

Note. F = Female, M = Male.

**Table 2:** Semi-structured interview schedule.

When did you or another family member begin to notice that something was happening to your child? What did you perceive as different in your child’s development? Why?
How did you feel at the time of the diagnosis? What thoughts did you have as you tried to understand that diagnosis? What did you do about it?
Where did you encounter the most difficulty in raising your child? Why?
Do you feel that your general health and well-being has worsened in recent years compared to the time of diagnosis? Why?
Which changes occurred in your family dynamics in the moment of diagnosis on a social, economic/work, and leisure level and as a couple or with the child’s other siblings or relatives? Have these changes been maintained in the adult stage of your children?
What is your child’s relationship with his/her other siblings like?
How much time do you spend on yourself every week? What do you do in that free time?
Do you have family or close people (neighbors and friends) who currently help you? Did you have this support at the time of the diagnosis?
What do you think about the care your child receives from the health system?
What support have the different care associations provided you?
What are your main concerns about your child?
What did you previously think about your child’s future and how do you imagine it now?
What aspects of your life and that of your child do you think could be improved?

**Table 3:** Main themes and codes after the initial analysis.

Changes Produced after the Diagnosis	Interpersonal Relationships	Physical and Emotional Well-Being as a Caregiver	Concerns about the Future
Changing residence/home	Little family support	Poorer physical health	Child’s place of residence
Adequacy of the home for the child with ID	Social distancing	Anxiety/worry	Economic expenses and employment
Abandonment of the carer’s job	Problems with partner	Lack of self-care and enjoyment	Physical health status of the child
Economic difficulties		Feeling of sorrow/grief	Having family and social support
Relationship with the siblings of the child with ID		Positive aspects	Happiness of the child

**Table 4:** Main quotes associated with Theme 3: Physical and emotional well-being as a caregiver.

Codes	Quotes
Worse physical health	*“In the middle of the night I have to get up 3 or 4 times to change him. Nights where I don’t sleep, nights where I sleep for the first 3 or 4 h and that’s it, or he/she starts to make noise, I can’t get back to sleep” (I03 Father).*
Anxiety	*“It’s a huge suffering when he gets sick, I tremble when he gets sick. I don’t want anything to hurt him, so he doesn’t get worse, he doesn’t suffer, and that worries me a lot…” (I12 Mother)*. *“For some time now I’ve had anxiety attacks, in fact, they were affecting my heart, I have heart problems, I take drugs for that. The anxiety is because of the fear that something will happen to him, that his health will deteriorate, that he will not have any friends…” (I16 Mother).*
Lack of self-care and enjoyment	*“Before we had friends, but not anymore. It’s not the same to have one child to having three, and to have a child with difficulties… because that means that you can’t do the same as you did before. Also, if nobody helps you... I couldn’t go out much before, nor now...” (I06 Mother)*. *“When you go with these children to places where families with children who are okay go, they don’t accept them. So, because they are not accepted, you pull back and make your own life, you end up alone without having any time for yourself or the marriage...” (I04 Mother).*
Feeling of sorrow/grief	*“Well, the truth is that the mood is sadder because of the care, yes... We’re not going to say no... [But,] physically, no, because the truth is that she’s truly autonomous... on a physical level it hasn’t affected me...” (I02 Mother)*. *“They’re never entirely accepted. There you have your thorn... The thorn that could’ve been more independent, more autonomous. Within a normality, I would like it to be like that, but it’s not. What can I do? It’s not like that. I feel sorrow and sadness because I’m not like I used to be, as I remember being, or how I remember my brother or my friend were” (I09 Father).*
Positive aspects	*“My sisters say that I’m getting better with her every day, they say ‘she’s helped you to live’, and I believe it might be true because I don’t stop, I’m more active” (I05 Mother)*. *“My son, within the limits of his disability, has changed everything for the better, the experience has been hard, but his love is very enriching without a doubt” (I11 Father),*
